# A systematic review and meta‐analysis of the impact of cornelian cherry consumption on blood lipid profiles

**DOI:** 10.1002/fsn3.2416

**Published:** 2021-06-21

**Authors:** Keyhan Mohammadi, Mahmood Alizadeh Sani, Elyas Nattagh‐Eshtivani, Shakila Yaribash, Jamal Rahmani, Behdad Shokrollahi Yancheshmeh, David Julian McClements

**Affiliations:** ^1^ Department of Clinical Pharmacy Faculty of Pharmacy Tehran University of Medical Sciences Tehran Iran; ^2^ Division of Food Safety and Hygiene School of Public Health Tehran University of Medical Sciences Tehran Iran; ^3^ Department of Nutrition Faculty of Medicine Mashhad University of Medical Sciences Mashhad Iran; ^4^ Faculty of Pharmacy Tehran University of Medical Sciences Tehran Iran; ^5^ Student Research Committee Department of Clinical Nutrition and Dietetics Faculty of Nutrition and Food Technology Shahid Beheshti University of Medical Sciences Tehran Iran; ^6^ Food Safety Research Center (salt) Semnan University of Medical Sciences Semnan Iran; ^7^ Department of Food Science University of Massachusetts Amherst Amherst MA USA

**Keywords:** cornelian cherry, flavonoids, lipid profile, phenolics, supplementation

## Abstract

Polyphenolic and flavonoid compounds are claimed to improve blood lipid profiles and to provide protective effects against cardiovascular disease. For this reason, we conducted a systematic review and meta‐analysis of studies that comprehensively investigated the effects of cornelian cherry supplementation on lipid profiles in rat models. Up to December 2020, 855 articles were screened, and finally, seven articles were selected as eligible for the meta‐analysis. This meta‐analysis revealed that cornelian cherry supplementation significantly decreased low‐density lipoprotein (LDL) (WMD = −6.38 mg/dl; 95% CI, −9.93 to–2.84; *p* < .001), triglyceride (TG) (WMD = −52.36 mg/dl; 95% CI, −80.50 to −24.22; *p* < .005), and cholesterol level (WMD = −37.16 mg/dl; 95% CI, −51.19 to −23.13; *p* < .005) in treated rats compared with control groups. A nonsignificant increase in high‐density lipoprotein (HDL) level was observed (WMD = 4.21 mg/dl; 95% CI, −3.25 to 11.66; *p* = .268). These results suggest that cherry supplementation may have health effects by modifying lipid profiles. However, there is a need for more well‐controlled human clinical trials to make more definitive conclusions about the potential health benefits of cherry supplementation.

## Graphical abstract

According to the importance of polyphenolic and flavonoid compounds in improving lipid profile and protective effects on cardiovascular disease, we conducted a systematic review and meta‐analysis of studies that comprehensively investigated the effects of cornelian cherry supplementation on lipid profile.

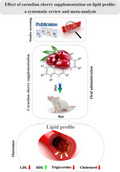

## 1 INTRODUCTION

Cardiovascular disease (CVD) is a leading cause of death globally that has significant effects on human health and the economy (Leal et al., 2006; Organization, 2014; Yusuf et al., 2001). The American Heart Association reported that 17.7 million people died from CVD in 2015, and this number is estimated to increase to around 23.6 million by 2030 (Mozaffarian et al., 2016). Dyslipidemia is one of the most important risk factors for CVD (Fakhrzadeh & Tabatabaei‐Malazy, 2012; McPherson et al., 2006; Rader, 2007). Dyslipidemia is defined by increased serum levels of triglycerides (TG), total cholesterol (TC), and low‐density lipoprotein cholesterol (LDL‐C), with reduced serum levels of high‐density lipoprotein cholesterol (HDL‐C) (Klop et al., 2013; Sahebkar & nutrition, 2017). Therefore, adopting a diet and lifestyle that can keep blood lipid profiles within the normal range is crucial for preventing CVD. Several lipid‐lowering pharmaceuticals can modulate blood lipid levels, with statins being one of the most widely used (Yan et al., 2006). However, there are concerns related to the long‐term use of statins because they can cause adverse side effects, such as myopathy and hepatotoxicity (Golomb & Evans, 2008; Harper & Jacobson, 2007). Thus, there is a growing interest in the use of natural substances with lipid‐modifying properties in combination with low‐dose statins, especially in individuals who are not able to tolerate high doses (Banach et al., 2018; Sahebkar et al., 2014; Sahebkar & Watts, 2014; Venero et al., 2010).

Consumption of fruits, particularly those rich in phenolics and flavonoids, has been claimed to improve blood lipid profiles and reduce the incidences of CVD (Mahmood et al., 2010; Zeng et al., 2012). Cornelian cherry (*Cornus mas*) contains many different kinds of biologically active phytochemicals, including tannins, phenols, organic acids, anthocyanins, ursolic acid, vitamin C, and flavonoids (Deng et al., 2013; Jayaprakasam et al., 2006; Seeram et al., 2002; Tural & Koca, 2008; Vareed et al., 2006). Numerous studies have demonstrated that cornelian cherries may exhibit a broad spectrum of biological activities, including antioxidant, antibacterial, anticancer, anticoagulant, antidiabetic, anti‐inflammatory, antiparasitic, lipid‐lowering, and cardio‐protective activities (Asgary et al., 2013; Asgary et al., 2014; Ghosh & Konishi, 2007; Jayaprakasam et al., 2006; Tural & Koca, 2008). A number of studies suggest that consumption of cornelian cherries may have beneficial effects on lipid disorders. For instance, it has been reported that consumption of 2 g/day of cornelian cherries decreased blood TG levels and increased HDL‐C levels in alloxan‐induced diabetic rats after a four‐week period (Asgary et al., 2014). Similarly, it has been reported that oral administration of an ethanol extract of cornelian cherry reduced TG, VLDL‐C, and LDL‐C levels, and increased HDL‐C levels in diabetic rats (Mirbadalzadeh & Shirdel, 2010). The ability of cornelian cherries to reduce blood TG levels after six weeks of intervention has also been shown in a clinical trial carried out on adults with type‐2 diabetes (Soltani et al., 2015). However, no significant effects of cornelian cherry consumption were reported on the blood lipid levels in postmenopausal women (Gholamrezayi et al., 2019). Therefore, the effect of cornelian cherry supplementation on lipid profiles is currently inconclusive, and there are inconsistencies between the results of different studies. To the best of our knowledge, there have been no previous systematic reviews and meta‐analyses of the effects of cornelian cherry supplementation on lipid profiles. For this reason, we carried out this analysis here, with a focus on the impact of cornelian cherry supplementation on the lipid profiles of rats.

## 2 METHODS

### 2.1 Data sources and search strategy

The Preferred Reporting Items for Systematic Review and Meta‐analysis [PRISMA] protocol was utilized to carry out a systematic review and meta‐analysis of the impact of cornelian cherry supplementation on blood lipid levels (Mohammadi et al., 2021; Moher et al., 2009). A detailed literature search was carried out using three online databases (Scopus, PubMed, and Embase) by two independent reviewers (MA, SY) from inception until December 2020 by using the following keywords: “LDL OR low‐density lipoprotein OR HDL OR high‐density lipoprotein OR TG OR triglyceride OR cholesterol OR lipids OR lipids profile OR lipid panel” AND “cornelian cherry OR cherry OR cornelian mass” AND “rat”. To avoid missing any relevant studies, we manually searched the references of the relevant review articles and the cited articles.

### 2.2 Study selection and inclusion criteria

Screening based on title, abstract, and keyword fields was conducted by two independent reviewers (EN, KM) to select relevant studies and then the full texts were screened to identify any cited studies that might also be relevant. The criteria used for inclusion were as follows: (a) the article was written in English; (b) the study evaluated the effects of cherry supplementation on the lipid profiles of rats; (c) at least one of the primary outcomes of the research included placebo and treatment groups. Thus, review articles, articles in other languages, studies that did not use rats as an experiment model, studies with unclear measurement units, and duplicate publications were excluded.

### 2.3 Data extraction

Data were selected and tabulated by the two reviewers (KM, MA) using a standardized data extraction form, developed according to the primary studies' variables. The data tabulated included the following: the first author's name, the publication year, the location of the study, the sample size, the type of rat used, the dose and duration of the treatment, and the mean ± standard deviation (*SD*) of the lipid profile markers at the end of the study. A third reviewer resolved any potential controversies. For those studies that reported the impact of cherry supplementation at more than one dose, each dose was treated as a separate study.

### 2.4 Statistical analysis

The effects of cherry supplementation on lipid profile markers were assessed by establishing and comparing the means and standard deviations using a weighted mean difference (WMD) approach at a confidence level of 95%. The overall magnitude of the effects was established utilizing a random‐effects model. The Cochran Q (P heterogeneity, significance level *p* < .1) and *I* ^2^ test were computed to determine potential heterogeneity among the included studies. Publication bias was established by carrying out Egger's regression test and by visual examination of the resulting funnel plot. Trim and fill analysis run on variables with significant publication bias. The sensitivity analysis was used to evolution the effect of each study on combined results. The potential impact of the dose of cherry supplementation was assessed by utilizing fractional polynomial modeling assuming a nonlinear dose–response relationship. All statistical analyses were performed using STATA statistical software version 16.0 (Stata Corporation).

## 3 RESULTS

### 3.1 Study selection

**FIGURE 1:**
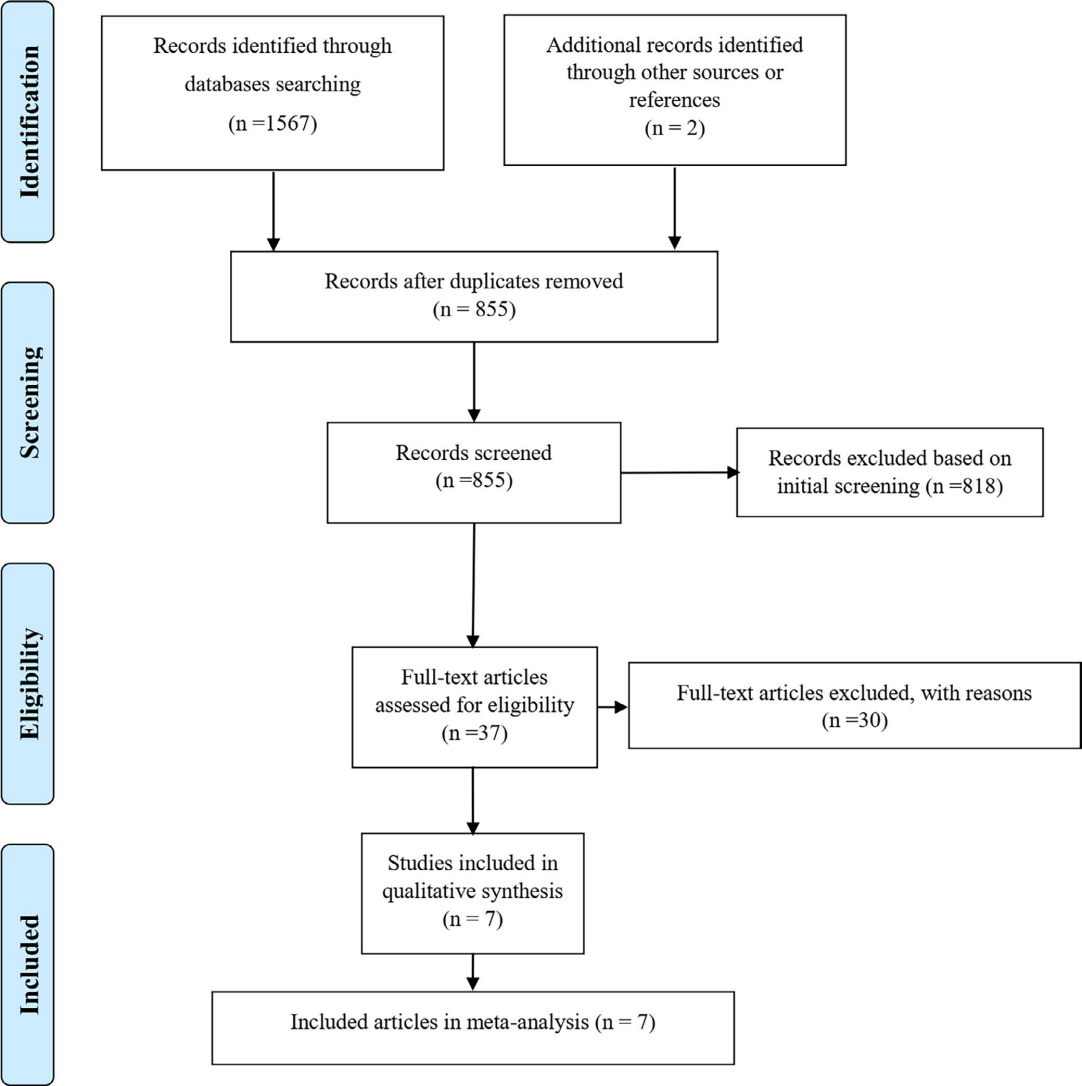
Preferred Reporting Items for Systematic Reviews and Meta‐analyses diagram indicating method for selection of papers included in the present study

A flowchart of the literature the search and selection procedure of the studies is presented in the PRISMA diagram (Figure 1). In the systematic search on Scopus, PubMed, Embase, and relevant studies from cross‐references, 1,567 articles were identified. After removing any duplicates, 855 articles were eligible for title/abstract screening and 55 articles remained for full‐text evaluation. Finally, seven animal studies were included in the final systematic review and meta‐analysis.

### 3.2 Studies characteristics

The main features of the included studies are shown in Table 1. Of these seven studies, five studies were conducted in Iran (Abdollahi et al., 2014; S. Asgary et al., 2014; Hosseinpour et al., 2017; Mirbadalzadeh & Shirdel, 2012; Vardin et al., 2017), one in Poland (Francik et al., 2017), and one in China (Yang et al., 2014). These studies were published between 2012 and 2017. Only the data obtained for rats were included in our animal analysis. The sample size in the included trials ranged from 20 to 54. The duration of cornelian cherry supplementation ranged from 10 to 42 days. In total, 622 data points were included in this analysis. The dose of cherry supplementation ranged between 0.05 and 20 g kg^−1^ day^−1^.

**TABLE 1:** Characteristics of included studies

Author/publication year	Country	Species tested	Sample size; intervention arms of measured outcomes	Sample size; control arm	Duration of exposure (days)	Intervention groups dose (g/kg)	Control group	Measured outcome(s)
Vardin et al., 2017	Iran	Wistar rats	24 (8, 8, 8/group)	16 (8, 8)	16	0.3, 0.7, 0.7	Distilled water	LDL, HDL, TG, Cholesterol
Francik et al., 2017	Poland	Wistar rats	54 (6/group)	6	35	10%	Fructose; high‐fat diet	HDL, TG, Cholesterol
Hosseinpour et al., 2017	Iran	Wistar rats	36 (6, 6, 6, 6, 6, 6/group)	12 (6, 6)	28	2.5, 5, 10, 10, 20	Basic diet (corn, soybean, wheat, rice bran)	LDL, HDL, TG, Cholesterol
Abdollahi et al., 2014	Iran	Wistar rats	24 (8, 8, 8/group)	8	21	0.05, 0.2, 0.4	Normal diet	LDL, HDL, TG, Cholesterol
Asgary et al., 2014	Iran	Wistar rats	14 (7, 7/group)	7	28	2	Normal diet	LDL, HDL, TG, Cholesterol
Yang et al., 2014	China	Sprague Dawley rats	5 (5/group)	5	42	8.13	Normal diet	LDL, HDL, TG, Cholesterol
Mirbadalzadeh et al., 2012	Iran	Wistar rats	10 (10/group)	10	10	0.1	Normal diet	LDL, HDL, TG, Cholesterol

### 3.3 Meta‐analysis results

#### 3.3.1 LDL level change

**FIGURE 2:**
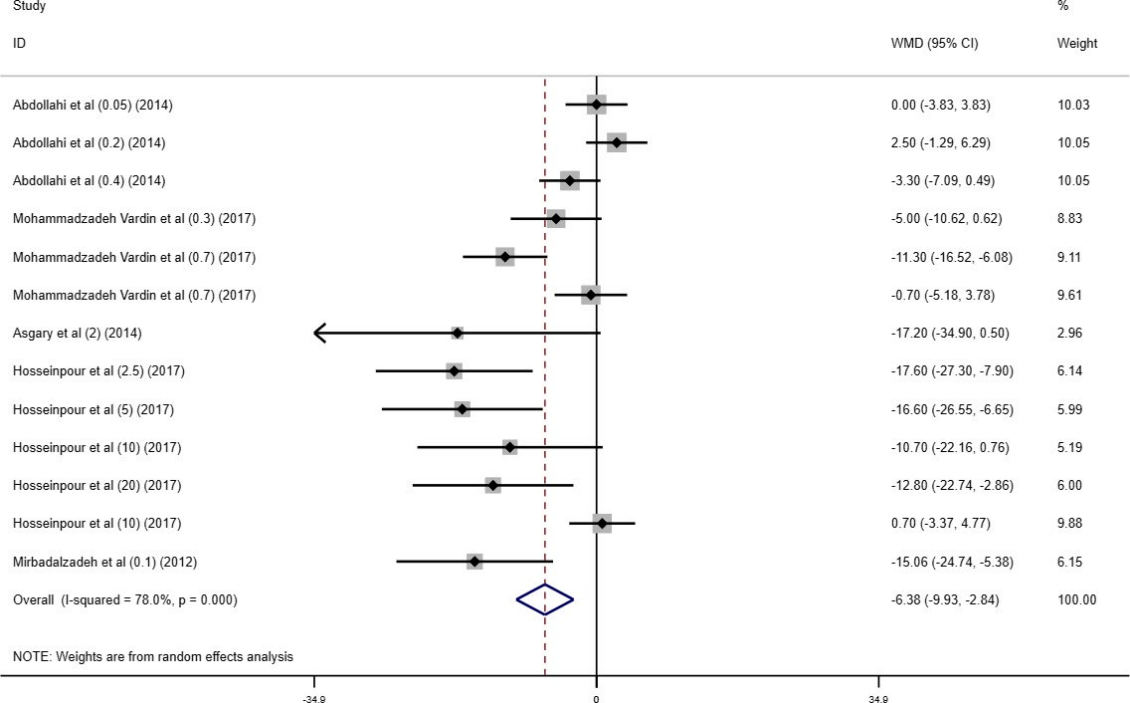
Forest plot presenting mean difference (WMD) and 95% CI for the effect of cherry supplementation on LDL levels

Combining the findings from 5 studies with 13 arms, data analysis indicated a significant reduction in serum LDL concentrations after cherry supplementation, as compared to the placebo (weighted mean difference [WMD]− 6.38 mg/dl; 95% CI (− 9.93, − 2.84), *p* < .001) with considerable heterogeneity among the included studies (*I* ^2^ = 78.0% *p*_heterogeneity_ < 0.001) (Figure 2).

#### 3.3.2 HDL level change

**FIGURE 3:**
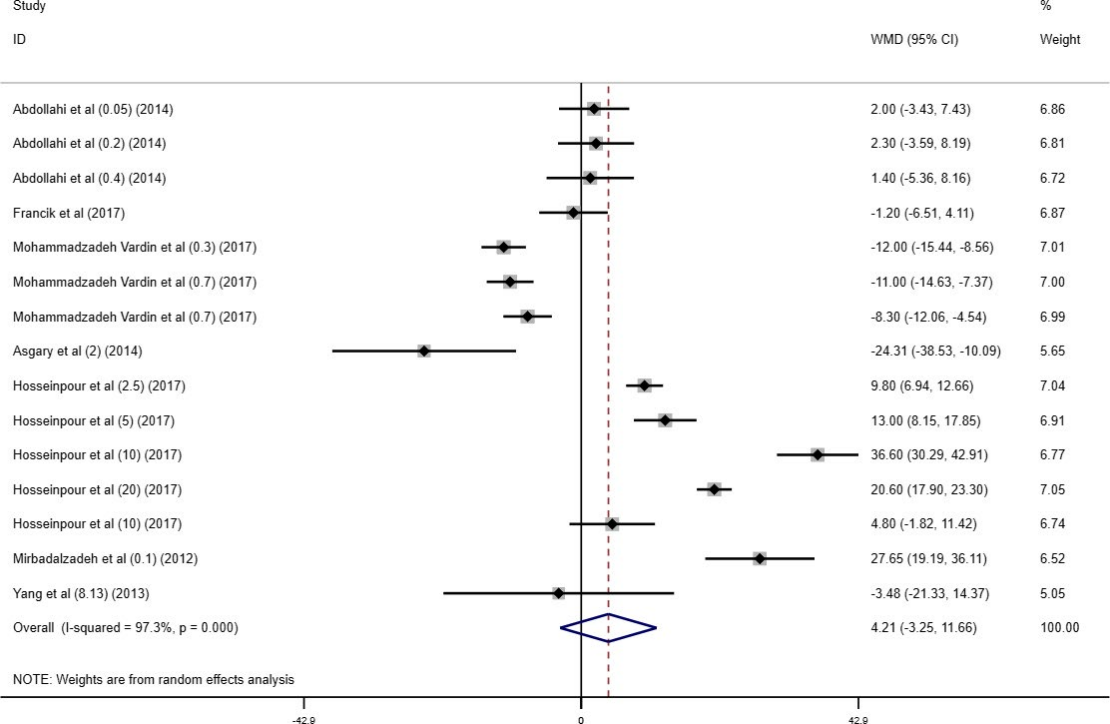
Forest plot presenting mean difference (WMD) and 95% CI for the effect of cherry supplementation on HDL levels

Seven studies including 15 arms reported changes in HDL blood levels as an outcome. A non‐significant increase in the HDL level following cherry supplementation was seen (WMD 4.21 mg/dl; 95% CI (− 3.25, 11.66), *p* = .268). However, a significant heterogeneity was observed (*I* ^2^ = 97.3%; *p*_heterogeneity_ < 0.001) (Figure 3).

#### 3.3.3 Cholesterol level change

**FIGURE 4:**
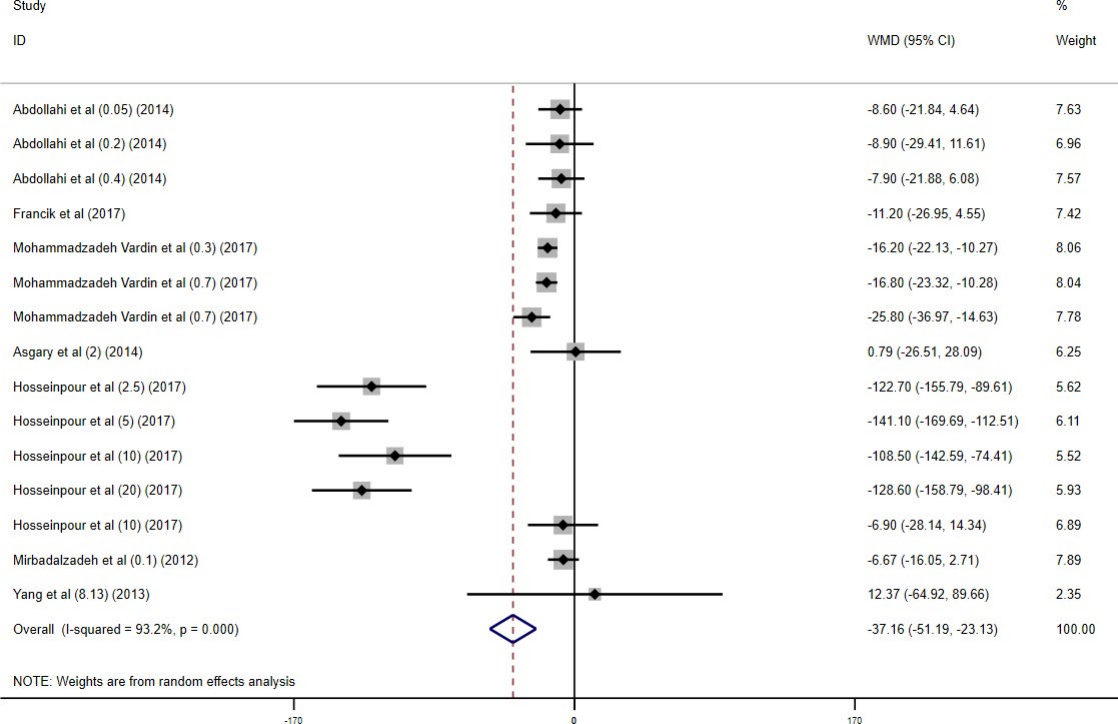
Forest plot presenting mean difference (WMD) and 95% CI for the effect of cherry supplementation on cholesterol levels

For evaluating the effect of cherry administration on cholesterol level, data from seven articles including 15 different arms were included in the analysis. A significant decrease in cholesterol level was observed following cherry supplementation (WMD −37.16 mg/dl; 95% CI (− 51.19, −23.13), *p* < .005) with significant heterogeneity across studies (*I* ^2^ = 93.2%; *p*_heterogeneity_ < 0.001), as shown in Figure 4.

#### 3.3.4 TG level change

**FIGURE 5:**
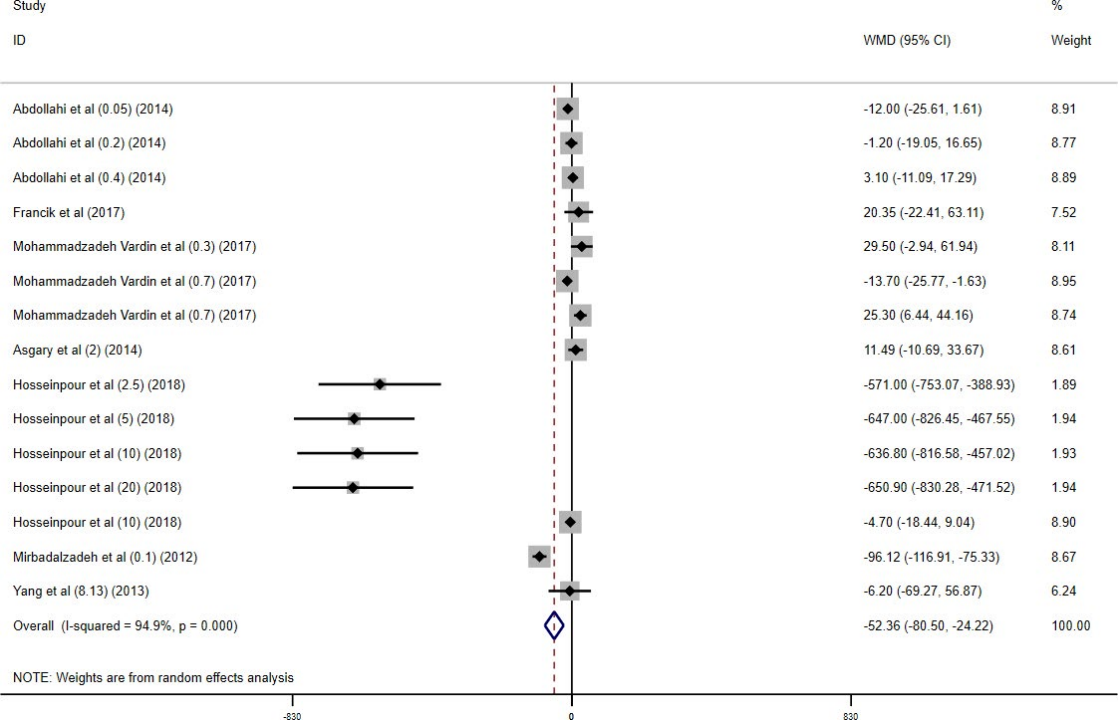
Forest plot presenting mean difference (WMD) and 95% CI for the effect of cherry supplementation on triglycerides levels

Application of the random‐effects model to the pooled results from seven studies with 15 arms showed an overall significant decrease in TG levels following cherry supplementation (WMD = −52.36 mg/dl; 95% CI, −80.50, −24.22; *p* < .005) with significant between‐study heterogeneity (*I* ^2^ = 94.9%, *P*_heterogeneity_ < 0.001) (Figure 5).

### 3.4 Dose‐response and meta‐regression

Meta‐regression analysis was utilized to evaluate the association between the duration of the intervention and the observed changes in blood lipid profiles. We carried out dose–response analyses using fractional polynomial modeling to find the nonlinear dose–response relationship between different administered doses of cherry (mg/kg) and change in lipid parameters level.

No significant association was observed when different durations of cherry supplementation were compared with alterations in LDL levels (Coefficient = −1.46, *p* = .585), HDL (Coefficient = .37, *p* = .924), cholesterol levels (Coefficient = −11.01, *p* = .403) and TG levels (Coefficient = −52.9, *p* = .420) (Figures S1–S4).

Following the dose–response evaluation, no significant association was observed between cherry dosage range (mg/kg) and changes in LDL, HDL, cholesterol, and TG levels in a nonlinear fashion (Figures S5–S8).

### 3.5 Publication bias, quality assessment of the included studies, trim and fill and sensitivity analysis

The results of Egger's tests of measured outcomes in the included studies were as follows: (LDL: *p* = .001, HDL: *p* = .840, cholesterol: *p* = .053, TG: *p* = .011) (Figures S9–S12). Trim and fill analysis run on LDL and TG in order to adjust for such biases (Table S1). The sensitivity analysis showed no statistically significant differences beyond the confidence interval of calculated combined results for each outcome (Figures S13–S16).

### 3.6 Discussion

Dyslipidemia is related to an increased risk for CVD, which causes millions of deaths globally every year. Therefore, therapeutic approaches based on dietary supplements or functional foods have been proposed to promote desirable blood lipid profiles and thereby prevent CVD. Among the supplements which have beneficial health effects, cornelian cherry has gained increasing attention as a lipid‐lowering agent.

Our meta‐analysis results show a significant association between the supplementation of the diets of rats with cherries and a reduction in TG, cholesterol, and LDL levels. A nonsignificant increase in HDL level was also noted in these animal studies. No significant association was found between change in lipid parameters when different doses and durations of the cherry supplements were administered.

It has been proposed that cornelian cherry supplementation may have health promoting effects because of its broad range of biological activities, including antioxidant, anti‐inflammatory, and lipid‐lowering effects (Szczepaniak et al., 2019). In particular, it is claimed that these biological activities may reduce cardiovascular disease, diabetes, and obesity, which has mainly been attributed to the presence of relatively high levels of certain polyphenolic compounds (Lietava et al., 2019). Studies have reported that fruits rich in anthocyanins, flavonoids, and phenolic substances, like cornelian cherries, have a strong antioxidant activity, which may contribute to their ability to reduce dyslipidemia by lowering TC and LDL‐C levels (Asgary et al., 2014; Hosseinpour et al., 2017; Mirbadalzadeh & Shirdel, 2010; Seymour et al., 2008; Zern et al., 2005). Several studies have been shown to have favorable effects on lipid profile. The lipid‐lowering effect of cornelian cherries may be due to their impact on glucose metabolism. Both animal and human studies have shown that supplementation with cornelian cherries can be an effective means of reducing blood glucose levels in those with hyperglycemia (Gholamrezayi et al., 2019; Soltani et al., 2015), increasing insulin sensitivity and improving insulin resistance (Gholamrezayi et al., 2019). Based on the observed results, it has been proposed that insulin resistance of the adipocytes can cause an increase in the release of fatty acids into the circulation. Elevated levels of free fatty acids reach the liver, where they induce the assembly and secretion of VLDL, which finally leads to hypertriacylglycerolemia (Ginsberg, 2000; Grundy, 1999). In addition, another possible triglyceride‐lowering effect may be owing to the blood glucose‐lowering effect of cornelian cherries. Consequently, the decrease in blood glucose levels leads to an increase in the concentration of cyclic AMP, which reduces the TG concentration in the blood (Sutherland & Robison, 1969; Wu et al., 2012). Hence, considering the valuable role of cornelian cherries in glucose homeostasis, it has been suggested that supplementation with this kind of cherry may reduce blood serum levels of triglycerides (Gholamrezayi et al., 2019; Soltani et al., 2015). Cornelian cherries are known to contain a range of phytochemicals, such as anthocyanins, flavonoids, phenolic acids, and tannins (Milenković‐Anđelković et al., 2015), which may exhibit inhibitory activities on HMG‐CoA reductase through their ability to bind and inactivate enzymes (Ademosun et al., 2015; Baskaran et al., 2015; Lee et al., 2003). Considering the high content of polyphenolic compounds in cornelian cherries, the inhibition of HMG‐CoA reductase activity seems likely. Lipoprotein lipase (LPL) has a major role in lipid metabolism, converted the triglycerides in lipoprotein particles into free fatty acids. The serum level of this enzyme is indicative of LPL production mainly in the adipocytes (Saiki et al., 2006) and is inversely associated with serum TG levels and positively correlated with HDL‐Clevels (Watanabe et al., 1999). In vitro studies have shown that flavonoids enhance the expression of LPL in adipose tissue and muscle cells (Fan et al., 2006). Moreover, studies have shown that high doses of phenolic compounds can prevent hyperlipidemia by increasing LPL activity (Koshy et al., 2001; Li et al., 2011).

The main strength of the current study was a comprehensive overview of the effects of cornelian cherry supplementary on blood lipid profiles for the first time and a review of its protective effects. All of the lipid profile indices (LDL, HDL, cholesterol, and TG) were investigated. Moreover, the relatively low publication bias of the included studies was another strength of our research. According to the positive effects described, there are some limitations and drawbacks to be addressed in the current meta‐analysis study, for example, the limited number of clinical and human trials, administration of various doses and different follow‐up periods in the included studies, the small number of studies available, and the low sample size of the included studies. Accordingly, additional studies should be considered to investigate the impact of various cherries on lipid profiles in human trials.

## 4 CONCLUSION

To summarize, the obtained findings from this systematic review and meta‐analysis of the effects of cornelian cherry supplementation on the blood lipid profile of animal studies showed that supplementation significantly reduced the LDL, TG, and cholesterol levels and increased the HDL levels compared with the control group. Therefore, these findings suggest that cherry supplementation may have lipid‐modifying properties, which might be due to the fact that these cherries are a good source of polyphenolic and anthocyanin compounds. However, long‐term, well‐designed human, and clinical studies should be conducted to further assess the impacts of cornelian cherry consumption on various health outcomes.

## CONFLICT OF INTEREST

The authors declare that there is no conflict of interest.

## Supporting information

App S1
