# Supplementary material for: A systematic review and meta‐analysis of the impact of cornelian cherry consumption on blood lipid profiles
Source: Food Sci Nutr. 2021 Jun 21;9(8):4629–38. doi: 10.1002/fsn3.2416 (PMC8358377; doi:10.1002/fsn3.2416)
Supplement: Supplementary file 1 — App S1 [file FSN3-9-4629-s001.docx]

**Supplementary data**

**Table S.1**: Result of Trim and fill analysis for evaluation of publication bias

| **Method** | **Pooled** | **95% CI** | | **Asymptotic** | | **No. of studies** |
| --- | --- | --- | --- | --- | --- | --- |
|  |  | Lower | Upper | z_value | p_value |  |
| LDL | -3.38 | -9.93 | -2.84 | -4.15 | 0.01 | 13 |
| TG | -52.36 | -80.50 | -24.22 | -3.65 | 0.01 | 15 |

**
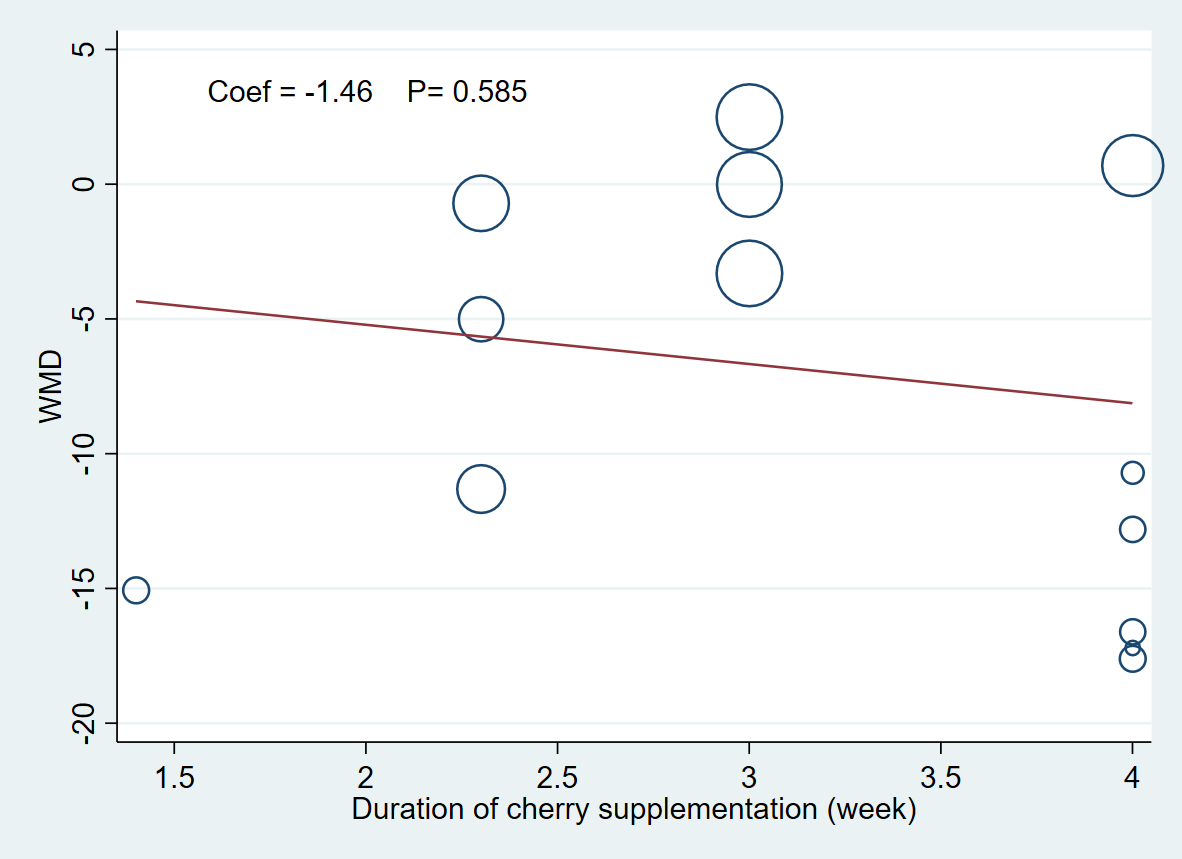
 Fig. S1.** Random-effects meta-regression plots of the association between WMD of LDL level change and cherry supplementation based on duration.

**
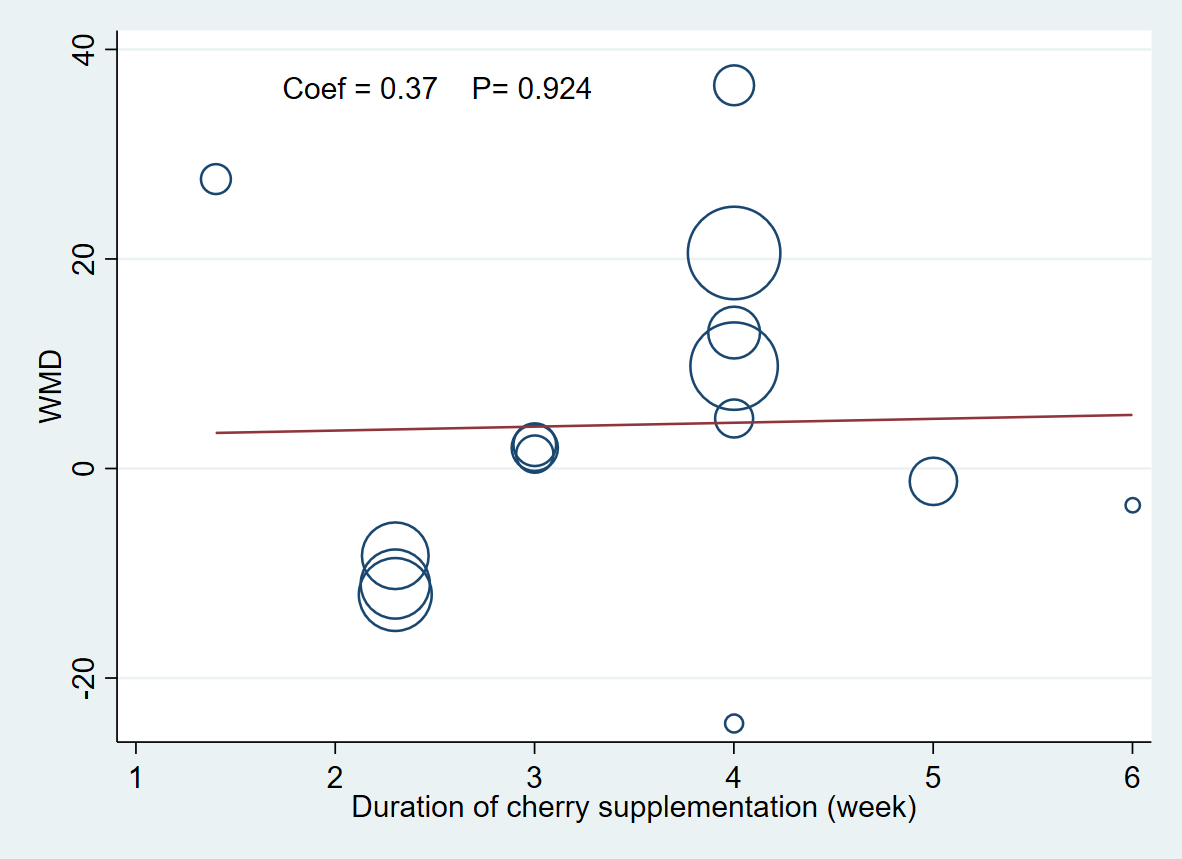
 Fig. S2.** Random-effects meta-regression plots of the association between WMD of HDL level change and cherry supplementation based on duration.

**
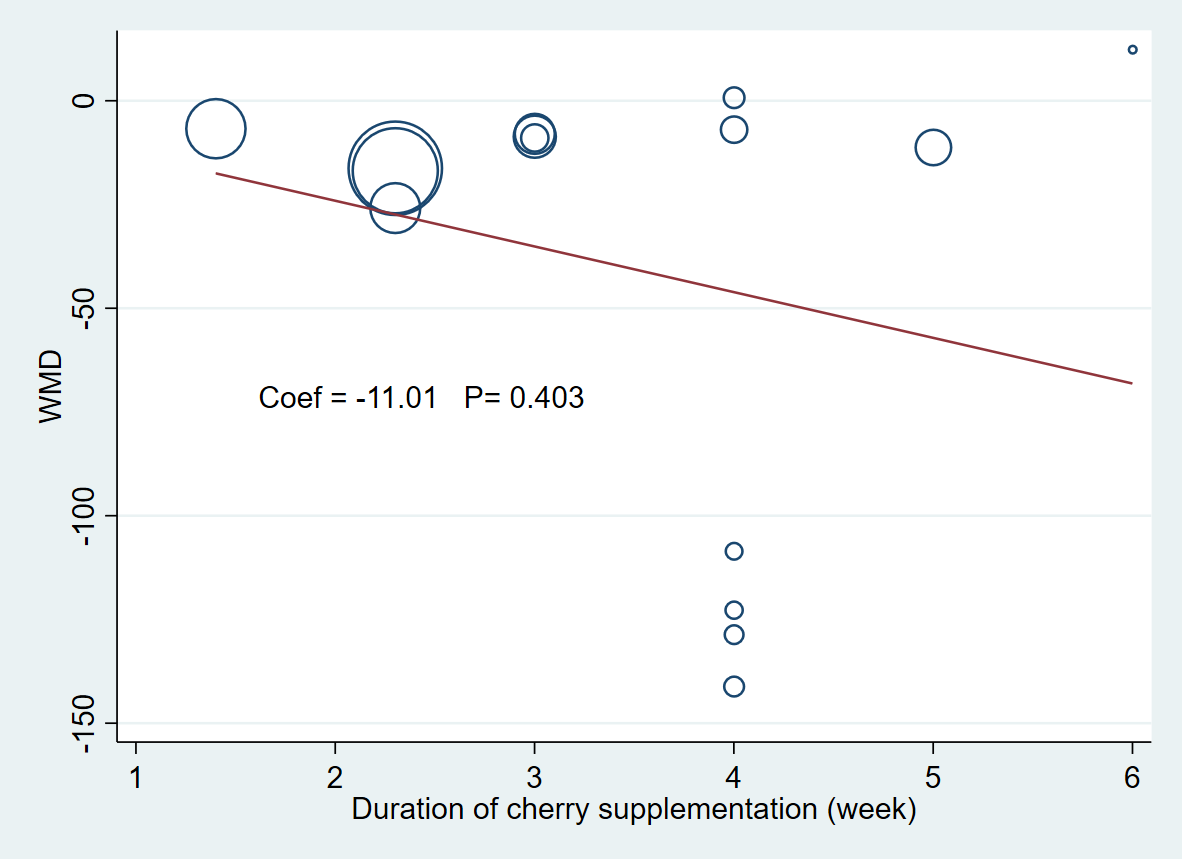
 Fig. S3.** Random-effects meta-regression plots of the association between WMD of cholesterol level change and cherry supplementation based on duration.

**
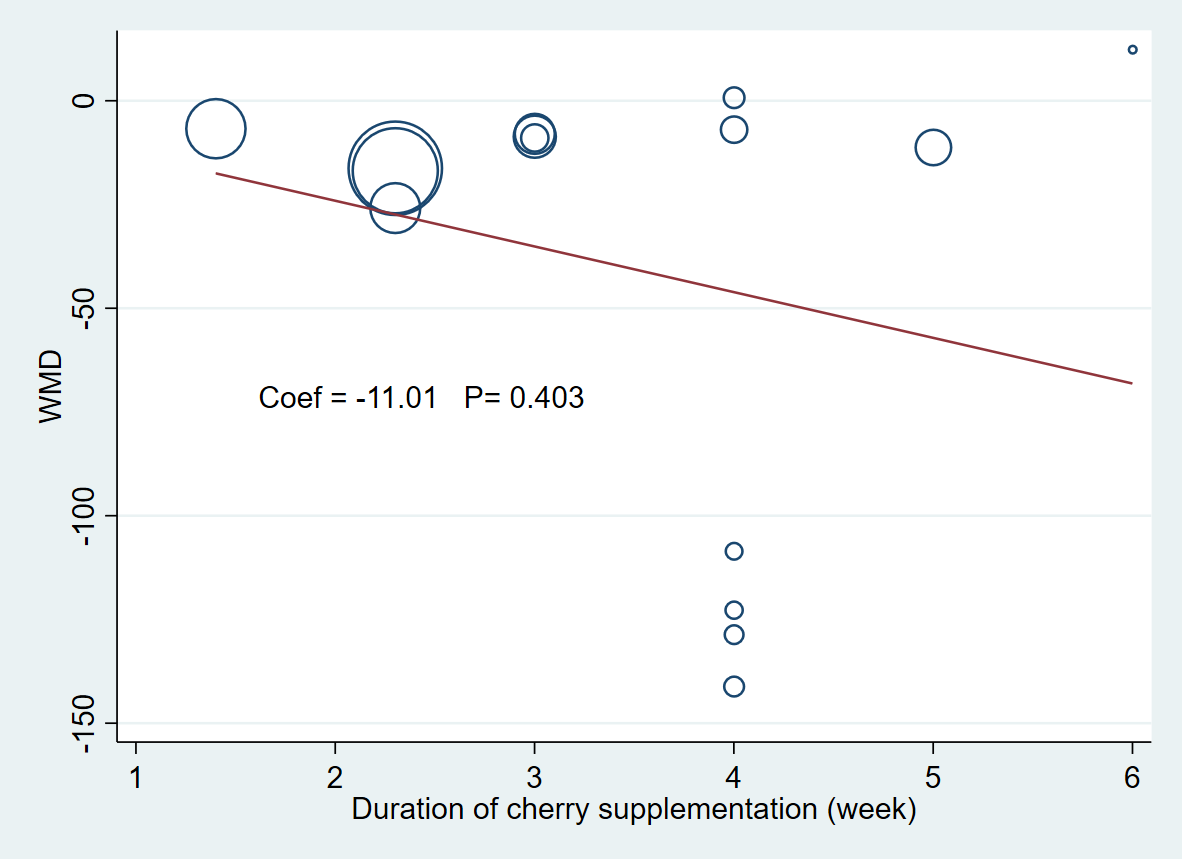
 Fig. S4.** Random-effects meta-regression plots of the association between WMD of triglycerides level change and cherry supplementation based on duration.

**
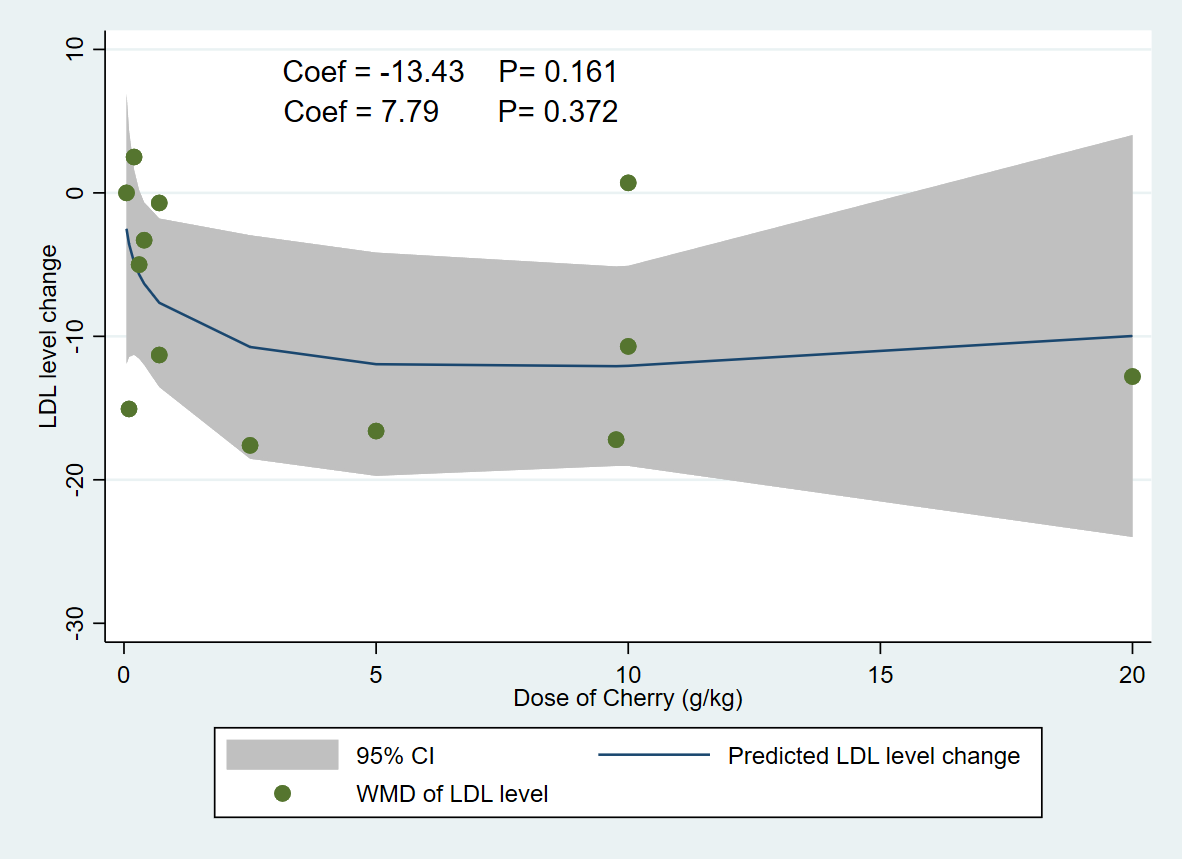
 Fig. S5.** Dose-response analysis between LDL level change and administered dose of cherry. The 95% CI is revealed in the shaded regions.

**
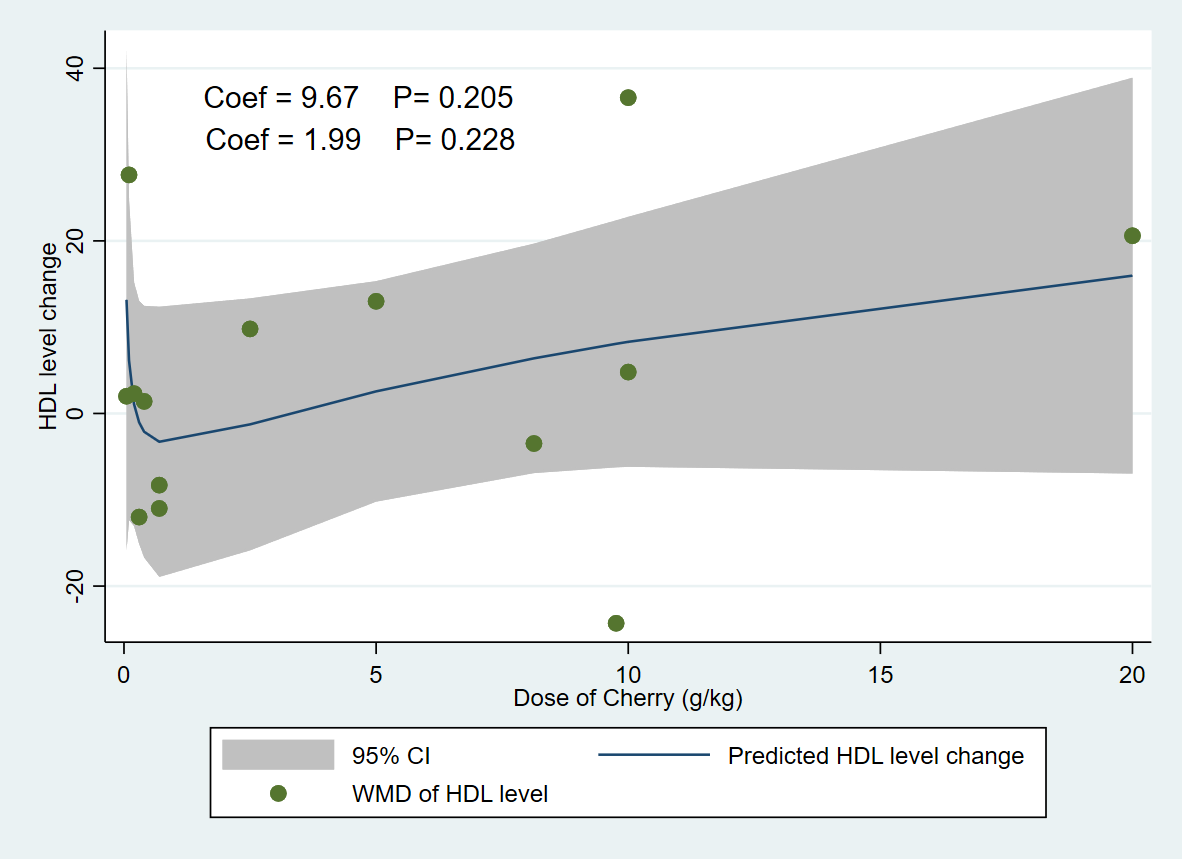
 Fig. S6.** Dose-response analysis between HDL level change and administered dose of cherry. The 95% CI is revealed in the shaded regions.

**
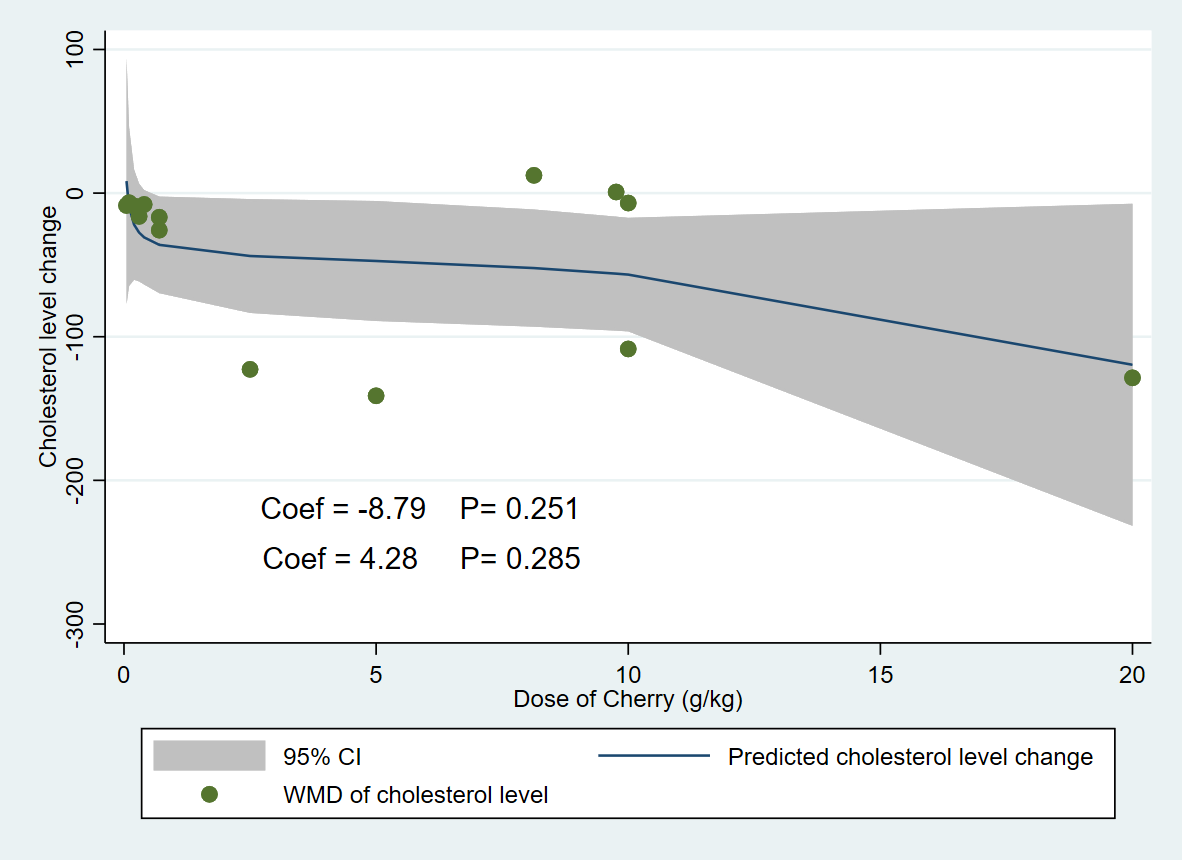
 Fig. S7.** Dose-response analysis between cholesterol level change and administered dose of cherry. The 95% CI is revealed in the shaded regions.

**
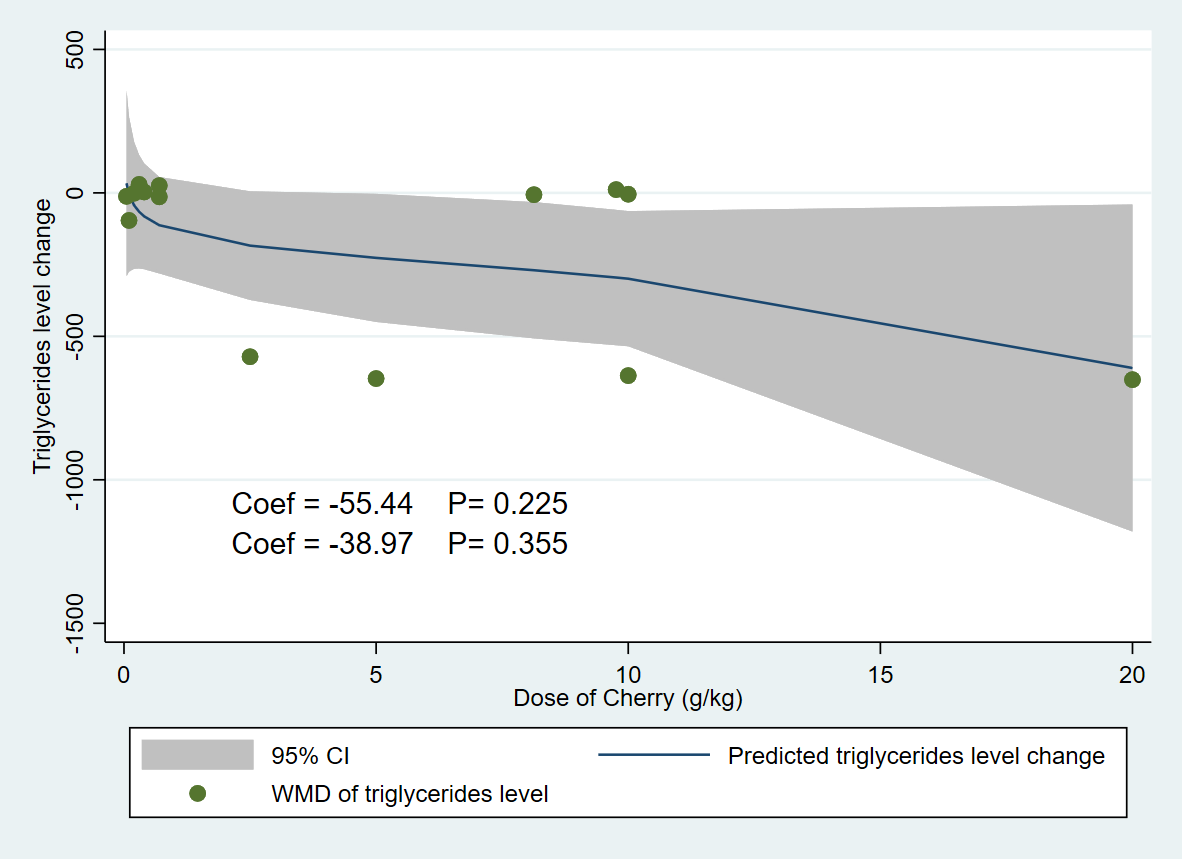
 Fig. S8.** Dose-response analysis between triglycerides level change and administered dose of cherry. The 95% CI is revealed in the shaded regions.

**
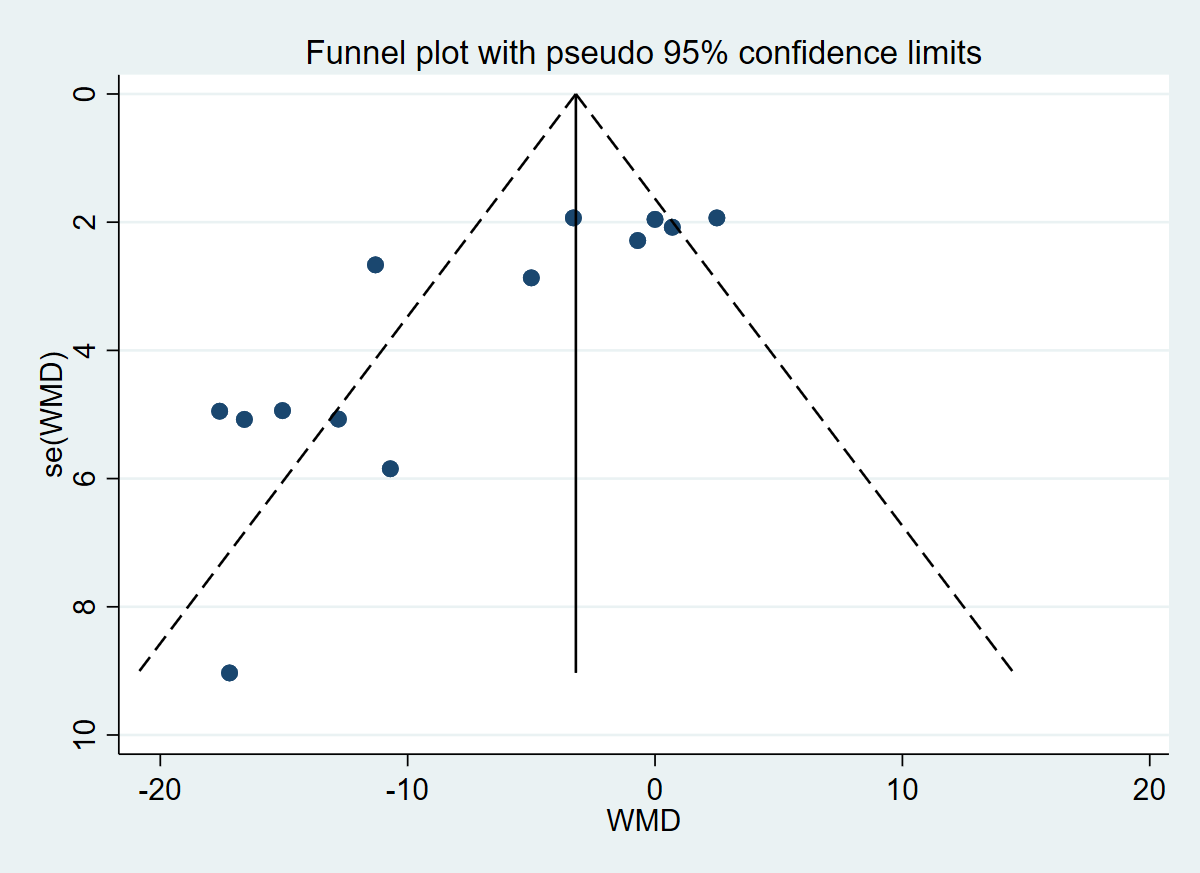
Fig. S9**. Funnel plot displaying publication bias in the studies reporting the impact of cherry supplementation on LDL level change.

**
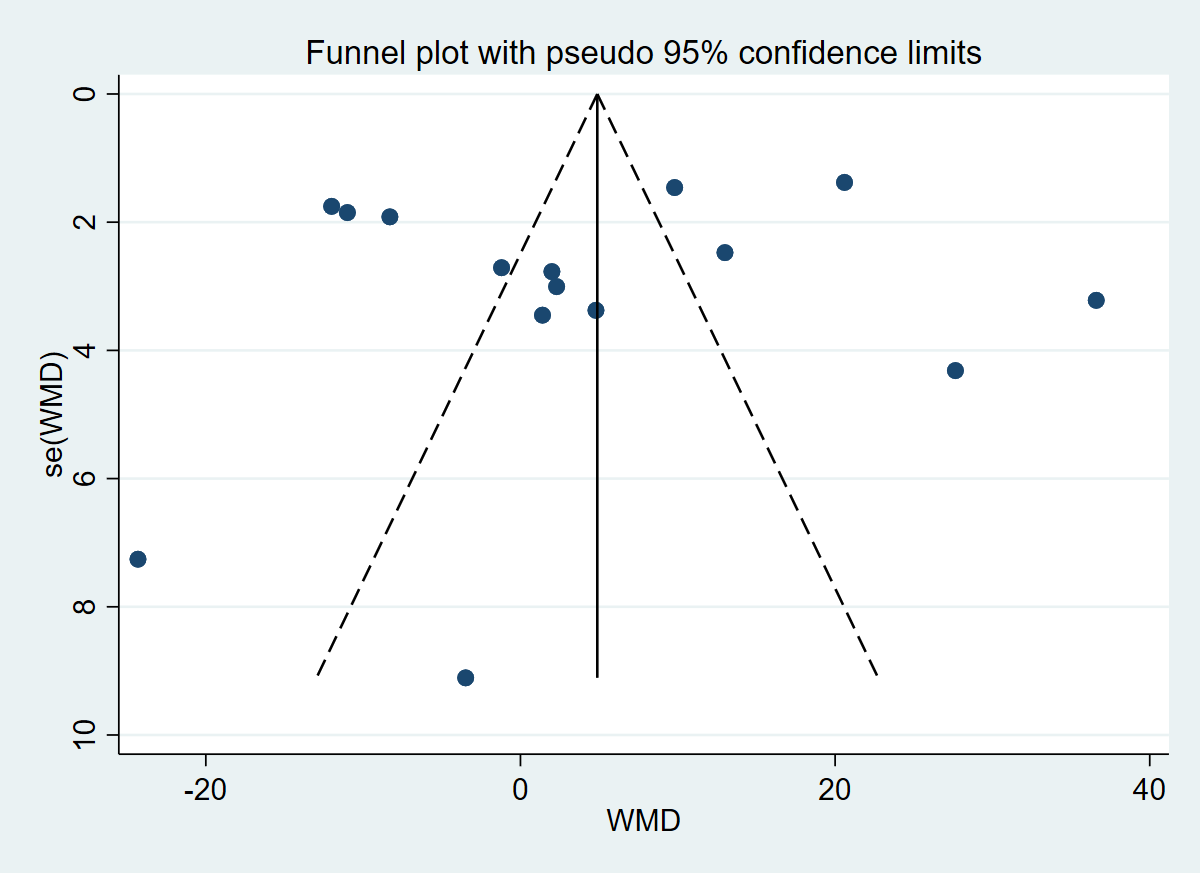
Fig. S10**. Funnel plot displaying publication bias in the studies reporting the impact of cherry supplementation on HDL level change.

**
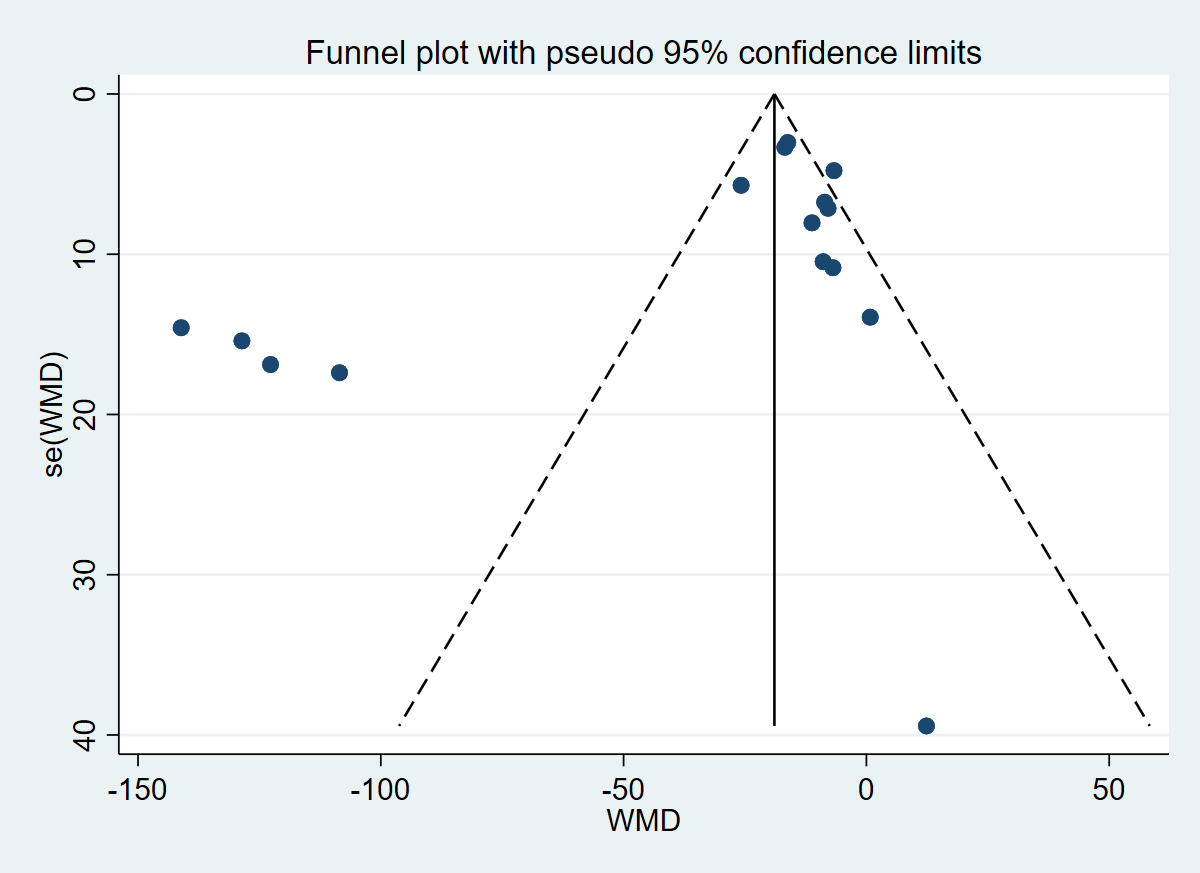
Fig. S11**. Funnel plot displaying publication bias in the studies reporting the impact of cherry supplementation on cholesterol level change.

**
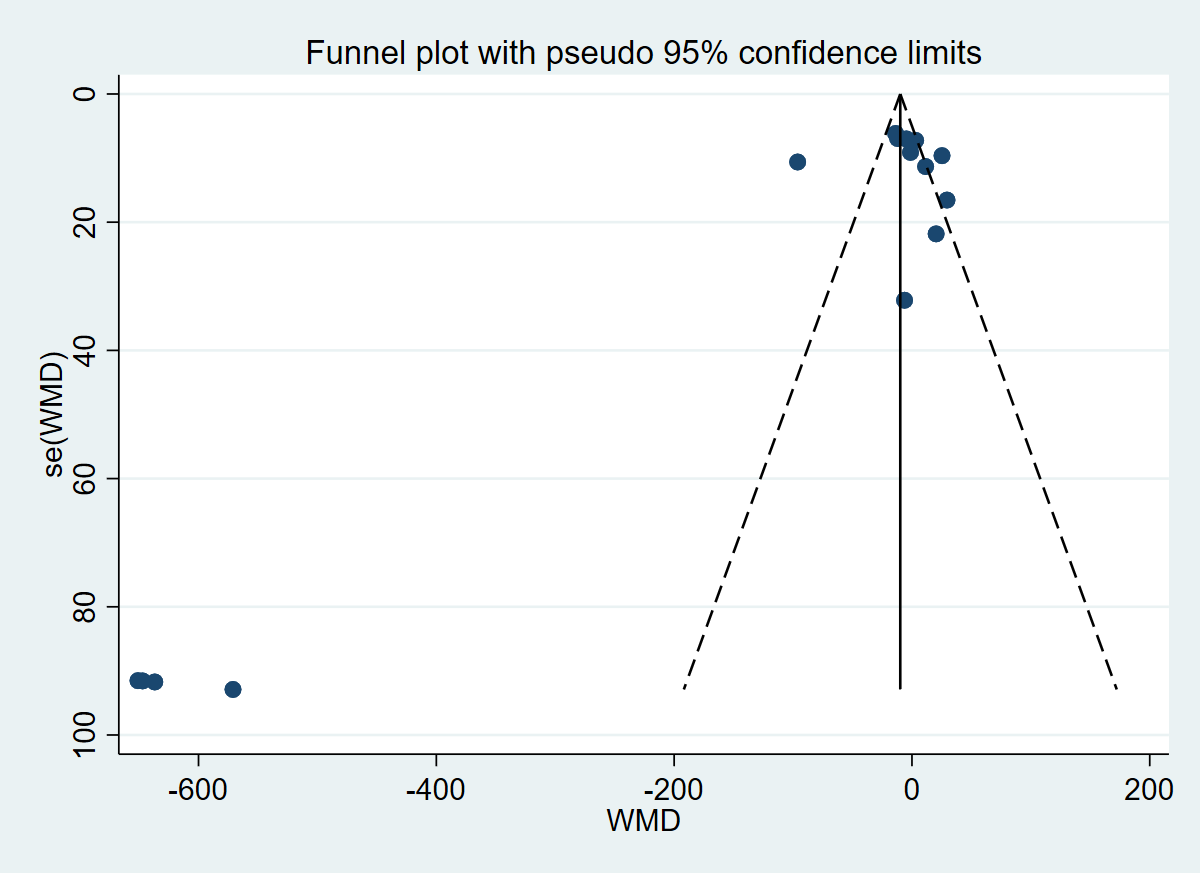
Fig. S12**. Funnel plot displaying publication bias in the studies reporting the impact of cherry supplementation on triglycerides level change.

**
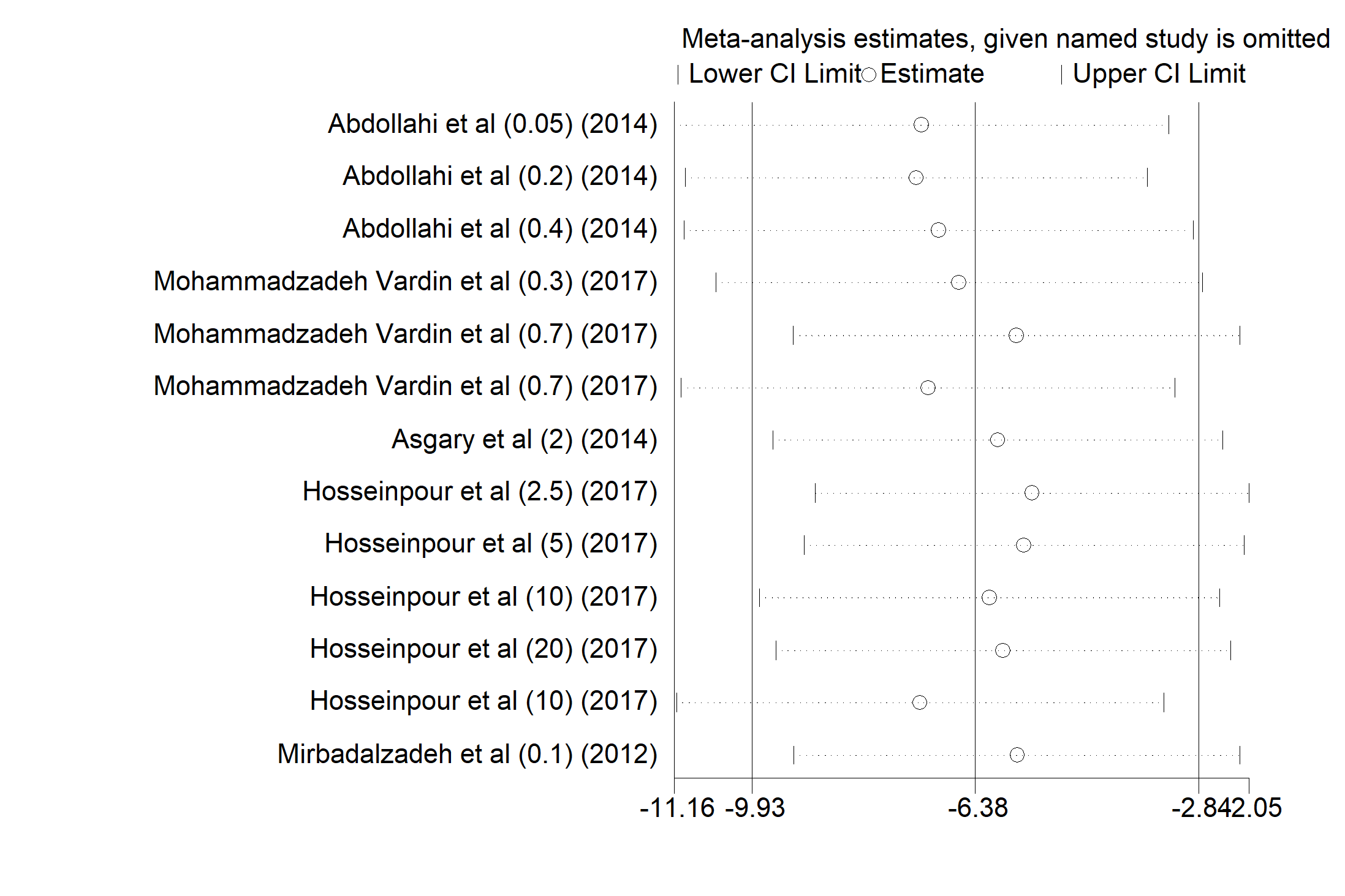
**

**Fig. S13**. The sensitivity analysis of calculated combined results for LDL outcome

**
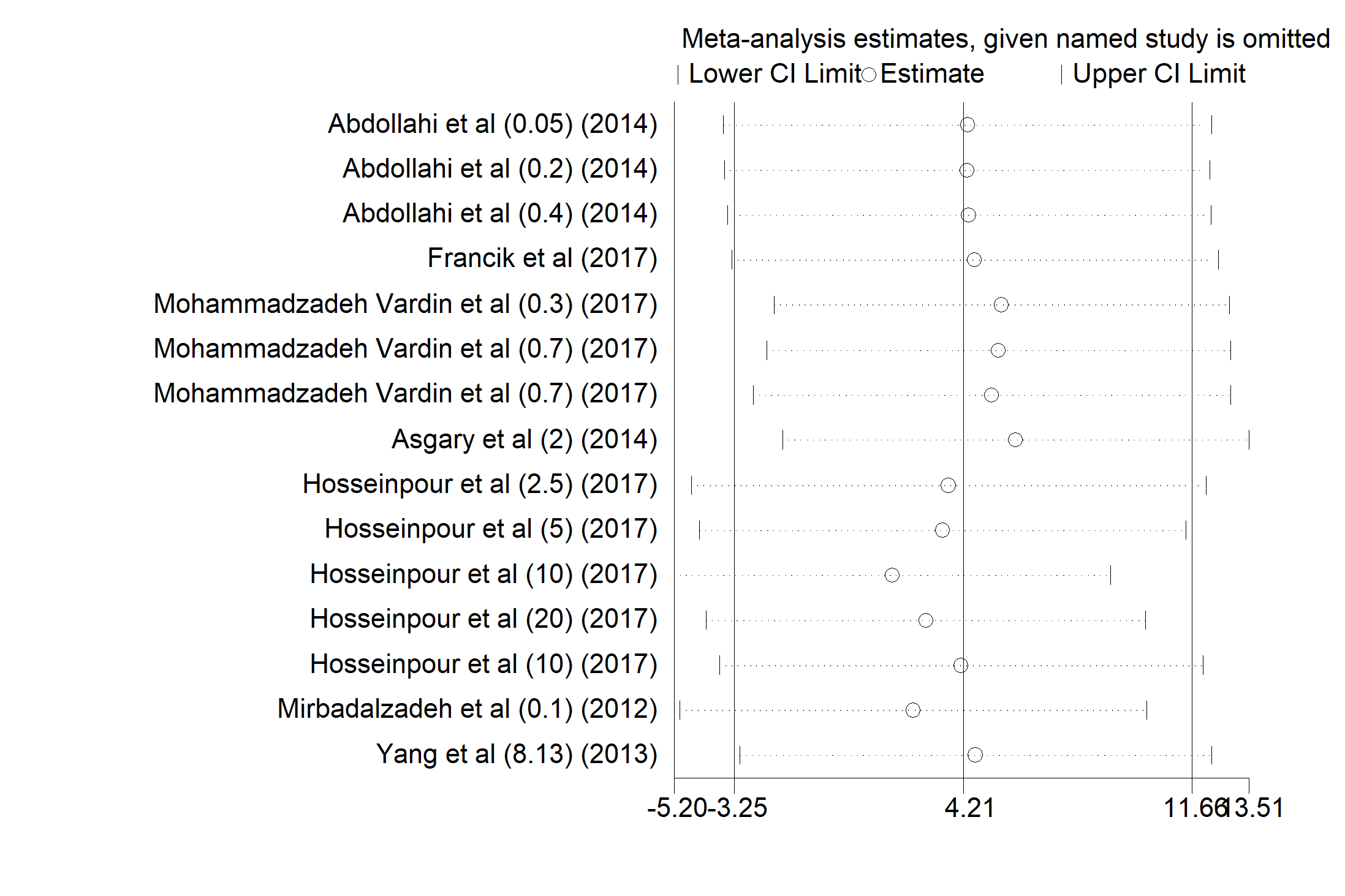
**

**Fig. S14**. The sensitivity analysis of calculated combined results for HDL outcome

**
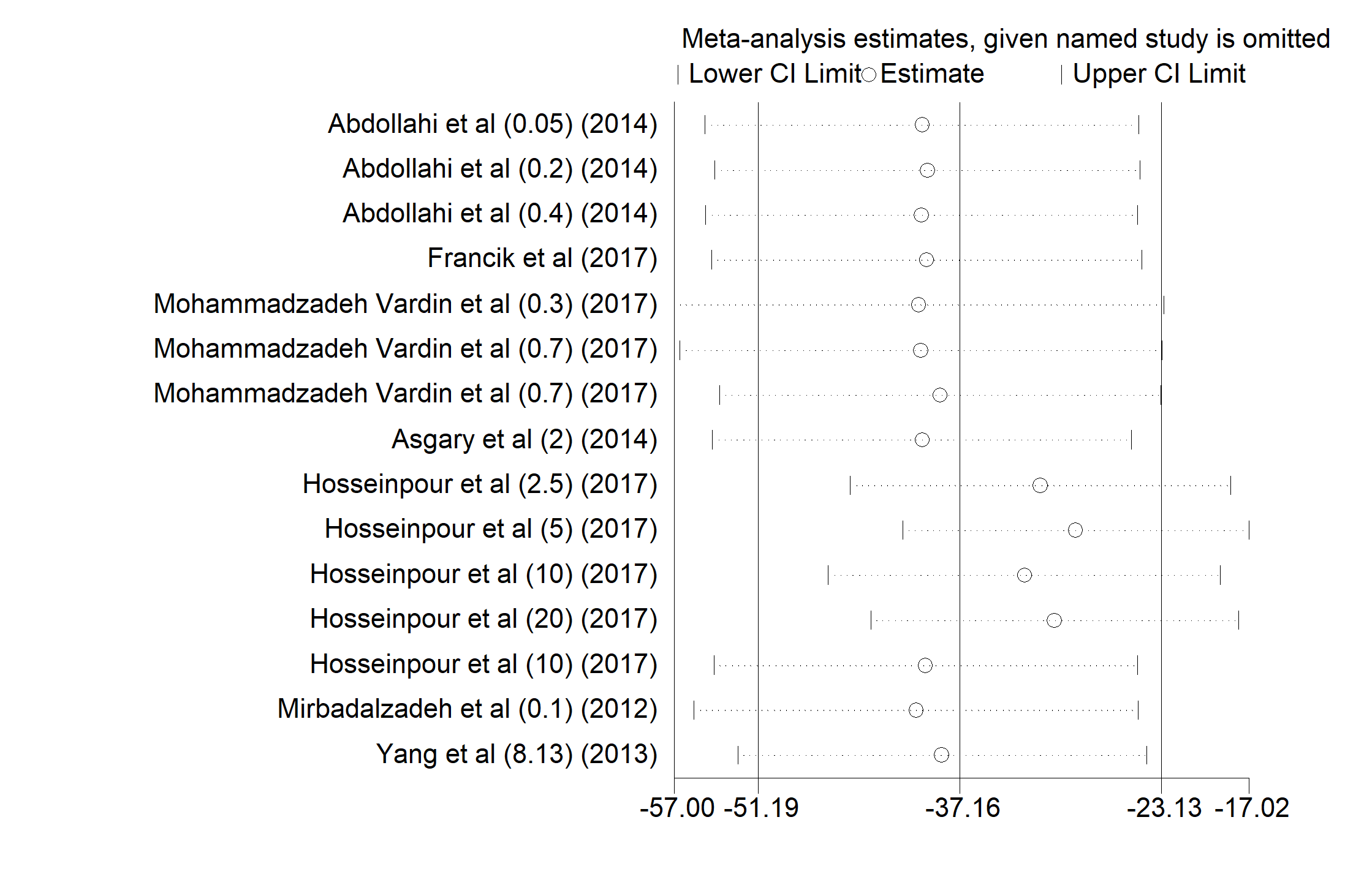
**

**Fig. S15**. The sensitivity analysis of calculated combined results for cholesterol outcome

**
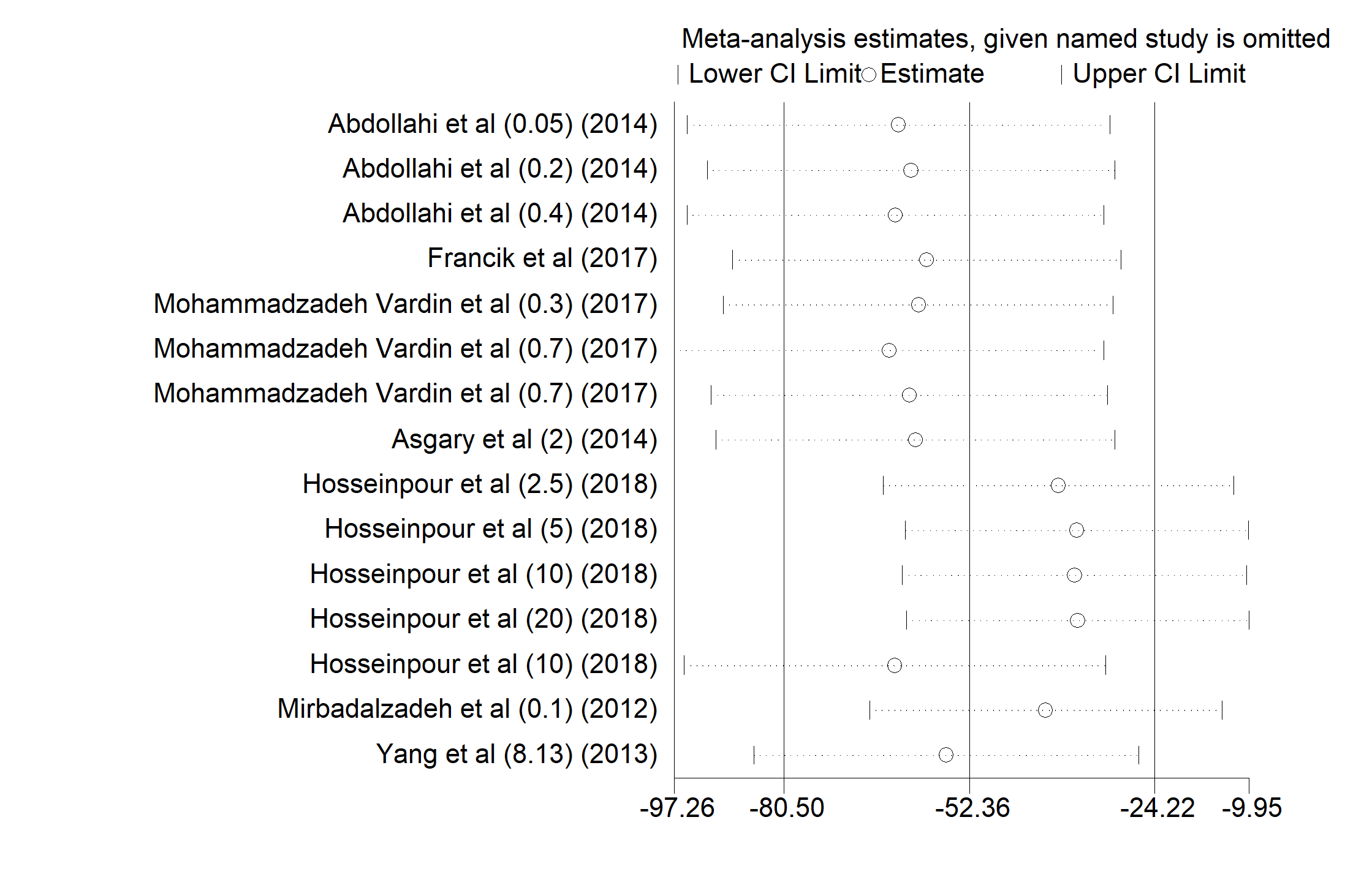
**

**Fig. S16**. The sensitivity analysis of calculated combined results for triglyceride outcome
